# Time-Course Transcriptome Study Reveals Mode of bZIP Transcription Factors on Light Exposure in *Arabidopsis*

**DOI:** 10.3390/ijms21061993

**Published:** 2020-03-14

**Authors:** Yukio Kurihara, Yuko Makita, Haruka Shimohira, Minami Matsui

**Affiliations:** 1 Synthetic Genomics Research Group, RIKEN Center for Sustainable Resource Science, Yokohama, Kanagawa 230-0045, Japan; yukio.kurihara@riken.jp (Y.K.); yuko.makita@riken.jp (Y.M.); haruka.shimohira@riken.jp (H.S.); 2 Graduate School of Nanobioscience, Department of Life and Environmental System Science, Yokohama City University, Yokohama, Kanagawa 236-0027, Japan

**Keywords:** *Arabidopsis*, Light, Transcriptome, *HY5*, bZIP

## Abstract

The etiolation process, which occurs after germination, is terminated once light is perceived and then de-etiolation commences. During the de-etiolation period, monochromatic lights (blue, red and far-red) induce differences in gene expression profiles and plant behavior through their respective photoreceptors. ELONGATED HYPOCOTYL 5 (HY5), a bZIP-type transcription factor (TF), regulates gene expression in the de-etiolation process, and other bZIP TFs are also involved in this regulation. However, transcriptomic changes that occur in etiolated seedlings upon monochromatic light irradiation and the relationship with the bZIP TFs still remain to be elucidated. Here, we track changes in the transcriptome after exposure to white, blue, red and far-red light following darkness and reveal both shared and non-shared trends of transcriptomic change between the four kinds of light. Interestingly, after exposure to light, *HY5* expression synchronized with those of the related bZIP TF genes, *GBF2* and *GBF3*, rather than *HY5 HOMOLOG* (*HYH*). To speculate on the redundancy of target genes between the bZIP TFs, we inspected the genome-wide physical binding sites of homodimers of seven bZIP TFs, HY5, HYH, GBF1, GBF2, GBF3, GBF4 and EEL, using an in vitro binding assay. The results reveal large overlaps of target gene candidates, indicating a complicated regulatory literature among TFs. This work provides novel insight into understanding the regulation of gene expression of the plant response to monochromatic light irradiation.

## 1. Introduction

Light is not only an essential source of energy production in plants but is also a key signal for the beginning of proper morphogenesis. Perception of light after germination is the first turning point for plants, because it triggers morphogenesis, including inhibition of hypocotyl elongation and expansion of cotyledons. This step is called photomorphogenesis [1]. Higher plants possess various kinds of photoreceptors, such as phytochromes, cryptochromes and phototropins; phytochromes perceive red and far-red light, while cryptochromes and phototropins respond to blue light [2]. Downstream signaling pathways after light perception are thought to be different between photoreceptors, but the signaling cascades from respective photoreceptors converge on a basic/leucine zipper (bZIP)-type transcription factor, ELONGATED HYPOCOTYL 5 (HY5) [3]. When diverse changes of gene expression occur in response to light exposure from darkness, expression of the *HY5* gene also responds dramatically to the light and accelerates downstream transcription for photomorphogenesis [4,5,6]. *Arabidopsis hy5*-defective mutants are strongly insensitive to light and the young seedlings show a long hypocotyl phenotype under light similar to plants grown in darkness [7,8,9,10,11]. Thus, HY5 acts as a central regulator of the light response and photomorphogenesis.

There are 78 bZIP family genes in the *Arabidopsis thaliana* genome [12,13]. As in the case of HY5, bZIP-type transcription factors (TFs) generally bind to G-boxes (CACGTG) with ACGT core sequences as homo- or hetero-dimers [14]. In addition to HY5, some other bZIP TFs are also known to be involved in the light response. HY5 HOMOLOG (HYH) promotes inhibition of hypocotyl elongation under light cooperatively with HY5 [9]. On the other hand, G-box binding factor 1 (GBF1), another bZIP TF with an N-terminal proline-rich region, acts antagonistically with HY5 and HYH in seedling development, probably by heterodimerization [9,15,16]. *GBF2* and *GBF3* were cloned together and also encode bZIP proteins with N-terminal proline-rich regions [14]. It has been reported that GBF2 localizes in the cytoplasm in the dark but moves into the nucleus in response to blue light, indicating its involvement in light signaling [17]. Although it is unknown whether GBF3 also acts in the light response, it is known to enhance ABA insensitivity and drought tolerance [18,19].

Chromatin immunoprecipitation combined with next-generation sequencing (ChIP-seq) has been used widely to establish in vivo genome-wide binding sites of TFs [20]. Recently, in vitro binding assays between recombinant TFs and purified or amplified fragments of genomic DNAs (gDNAs) have been developed (gDNA binding sequencing, gDB-seq, or DNA affinity purification sequencing, DAP-seq). The bound gDNA fragments are sequenced to easily identify genome-wide physical binding sites of TFs as a substitute method for ChIP-seq [6,21,22]. The results largely trace the binding sites identified by ChIP-seq and ChIP-chip analyses.

At present, transcriptomic transitions upon monochromatic light irradiation and the part played by bZIP TFs in the light response are still to be elucidated. Here, we report time-course transcriptome analysis upon light exposure and examine the association of some bZIP TFs. We believe that our results will help to expand the knowledge of the monochromatic light response.

## 2. Results

### 2.1. Time-Course Transcriptome during Light Exposure after Darkness

**Figure 1:**
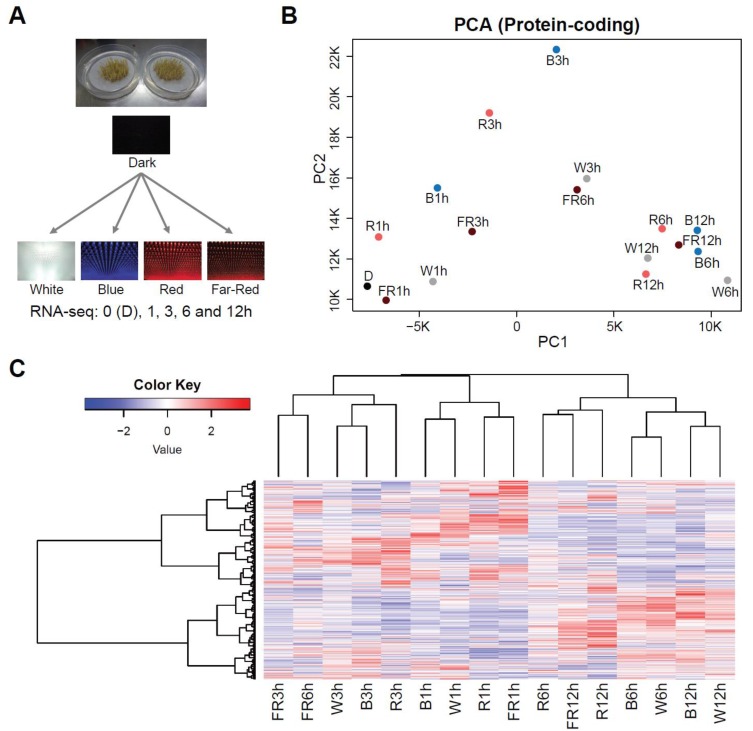
Time-course transcriptome analyses after light exposure from darkness. (**A**) Schematic explanation of the experiments performed in this research. Seedlings grown in the dark for 3 days were exposed to white, blue, red, or far-red light. RNA-seq analyses were performed at 0, 1, 3, 6 and 12 h after light exposure. (**B**) Principal component analysis for mRNA accumulation on exposure to light. W = White, B = Blue, R = Red, FR = Far-Red. (**C**) Heatmap (Z-score data) of fold changes (light/dark) of mRNAs that were detected by the time-course RNA-seq analysis.

To elucidate the time course of the transition in gene expression on exposure to white, blue, red and far-red light, we tracked changes in mRNA abundance by RNA-seq at time points (0, 1, 3, 6 and 12 h) after dark-grown 3-day-old seedlings were exposed to each light, as shown in Figure 1A.

Principal component analysis (PCA) indicated an apparent difference in gene expression between the four kinds of light up to 3 h after light exposure and thereafter expression converged (Figure 1B). Clustering analysis using whole gene expression profiles showed an apparent time-dependent similarity in the profiles of the four kinds of light (Figure 1C). This analysis also indicated that red and far-red light, rather than blue light, were relatively closely clustered. This result is consistent with the fact that both red and far-red light pass through the same photoreceptors and blue light is perceived through different ones [2].

### 2.2. Large Overlap of Differentially Expressed Genes (DEGs) between Three Monochromatic Light Exposures

Blue, red and far-red monochromatic light pass through their respective photoreceptors but may affect gene expression in a similar manner, because the signaling cascades meet each other at *HY5* [3]. To understand the commonalities and differences in gene expression induced by blue, red and far-red light exposure, we compared differentially expressed genes (DEGs), which were derived from 1 h or 3 h exposure. DEGs found only 6 h and 12 h after exposure were excluded from this analysis because it was unknown as to whether they were controlled by light or other factors, such as circadian rhythm.

**Figure 2:**
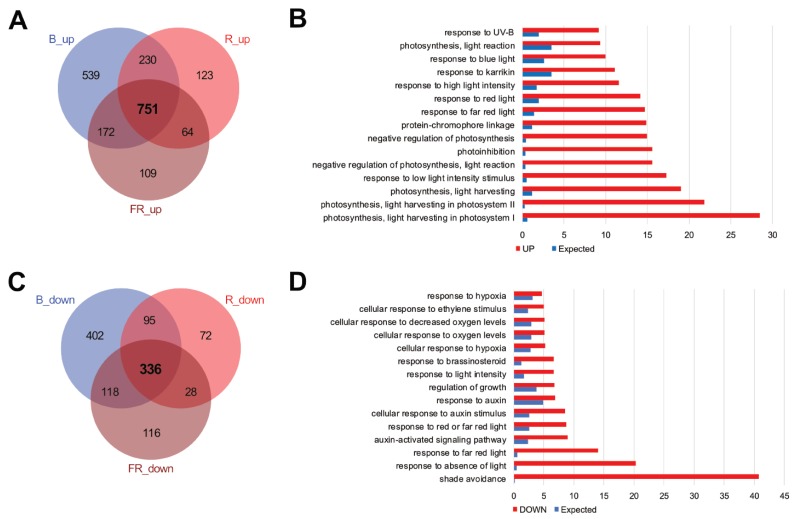
Comparison of differentially expressed genes (DEGs) in seedlings after the blue, red, and far-red monochromatic light irradiation. (**A**) and (**B**) Overlaps of up-regulated (**A**) and down-regulated (**B**) genes after the different monochromatic light irradiations. (**C**) and (**D**) Gene ontology (GO) term enrichments of up-regulated (**C**) or down-regulated (**D**) genes after the different monochromatic light irradiations. DEGs were derived from either 1 h or 3 h light exposures in RNA-seq analysis.

*Arabidopsis* has 27,655 protein-coding genes. At 1 h and 3 h after exposure, 1692, 1168 and 1096 up-regulated genes and 951, 531 and 598 down-regulated genes were identified in seedlings after blue, red and far-red light exposure, respectively (Table S1 and Figure S1). Of these, 751 and 336 genes were significantly up-regulated and down-regulated, respectively, in the three light conditions (Figure 2A,C). Gene ontology (GO) enrichment analysis, as expected, showed that light response and photosynthesis-related terms were enriched in the up-regulated genes, while dark-related terms were enriched in the down-regulated genes (Figure 2B,D).

The overlap of up-regulated genes (Figure 2A) was equivalent to 44.4%, 64.3% and 68.5% of DEGs in blue, red, and far-red light, respectively. We detected a relatively large number of independent DEGs (55.6%) in blue light. GO analysis of these showed that abiotic stress-related terms are enriched (Figure S2).

### 2.3. Association between HY5 Binding and Transcriptomic Changes

**Figure 3:**
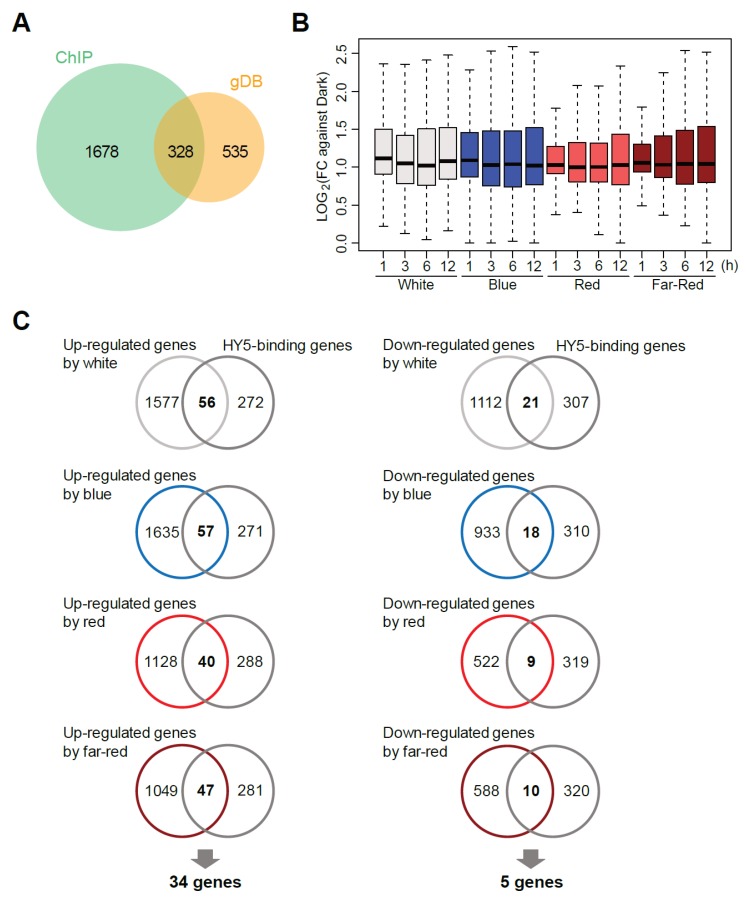
Relationship between ELONGATED HYPOCOTYL 5 (HY5)-binding genes and DEGs upon light exposure. (**A**) Overlap of the genes with HY5-binding sites in the promoters, which were identified by two methodologies, ChIP-seq and gDB-seq. (**B**) Boxplot of fold changes (light/dark) of the 328 HY5-binding genes which overlapped in (**A**). (**C**) Overlap between up-regulated or down-regulated genes and the 328 HY5-binding genes.

To understand the molecular basis of the transcriptional regulation, we re-analyzed the published genome-wide HY5-binding sites by in vivo ChIP-seq and in vitro gDB-seq analyses [6,23]. HY5-binding sites in the promoter regions of 328 genes were detected in both analyses (Figure 3A). The binding was loosely associated with early transcriptional induction of genes during exposure to all the types of light (Figure 3B), suggesting HY5 may function in the transcription of these mRNAs. However, the influence of HY5 on transcription may be limited because overlaps between the 328 HY5-binding genes and the DEGs upon light exposure were up to 17.4% (Figure 3C). Of the overlapped genes, 34 and 5 HY5-binding genes were up-regulated and down-regulated, respectively, in all light conditions, indicating that they are direct HY5 target genes (Figure S3).

### 2.4. Expression of HY5-Related bZIP TF Genes

**Figure 4:**
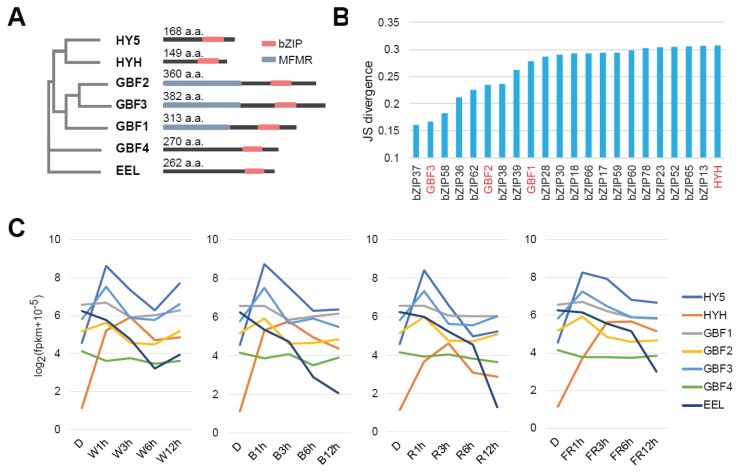
Expression profiles of bZIP transcription factor (TF) genes upon light exposure. (**A**) Phylogenetic tree and domain structures of HY5 and the other bZIP TFs. MFMR = MultiFunctional Mosaic Region. (**B**) Jensen-Shannon (JS) divergence of bZIP TF genes for co-expression with *HY5* on exposure to light. (**C**) Expression of seven bZIP genes, *HY5*, *HYH*, *GBF1*, *GBF2*, *GBF3* and *EEL*, in each type of light.

It is speculated that not only HY5 but also some bZIP TFs like HYH and GBF1 are involved in the light response, as described previously [9,15,16]. Here, we selected seven bZIP TF genes, *HY5*, *HYH*, *GBF1*, *GBF2*, *GBF3*, *GBF4* and *ENHANCED EM LEVEL* (*EEL*) for the following analysis (Figure 4A). The molecular features of GBF4 have been characterized previously [24]. It was reported that EEL, the amino acid sequence of which is relatively similar to GBF4, is involved in seed maturation [25], but there has been no report supporting the involvement of GBF4 and EEL in the light response. Here, GBF4 and EEL were selected as controls possibly unrelated to light response.

We examined and classified time-course expression changes of all the detected bZIP genes and found that the expression of *HY5*, *GBF1*, *GBF2* and *GBF3* was classified into close branches of the heatmap dendrogram (Figure S4 and Table S2). Additionally, co-expression analysis suggested that the expression profile of *GBF3* was the closest to that of *HY5* of the seven selected bZIP genes (Figure 4B,C). Interestingly, the expression profile of *HYH* was relatively distant from *HY5* (Figure 4B and Figure S4) and its maximum accumulation after each light exposure occurred later (Figure 4C).

No significant change in *GBF4* was detected under all light exposures and expression of *EEL* dramatically reduced after light irradiation (Figure 4C and Figure S4). This may mean there is a gradual reduction in seed-related expression with light-induced growth.

### 2.5. Binding Potential of bZIP TFs

**Figure 5:**
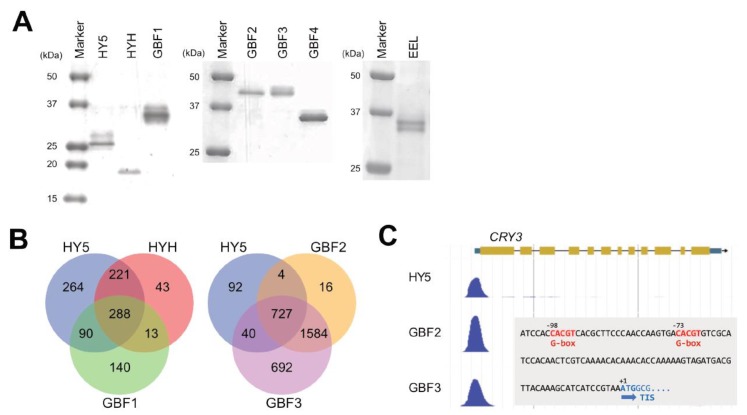
Comparison of physical binding sites identified by gDB-seq analysis among bZIP TFs. (**A**) Detection of recombinant proteins with an HA-tag at the N-terminal by SDS-PAGE gel electrophoresis. Recombinant proteins were synthesized in wheat germ extracts. (**B**) Overlap between genes with promoters possessing HY5-binding sites and genes with promoters possessing binding sites of HYH, GBF1, GBF2 or GBF3. (**C**) Nucleotide numbers are relative to the translation initiation site (TIS) (+1).

To know how many binding sites of the bZIP TFs overlap with those of HY5, we applied gDB-seq analysis to HYH, GBF1, GBF2, GBF3, GBF4 and EEL. For this purpose, recombinant TFs were synthesized using a wheat germ cell-free system (Figure 5A). These TFs were mixed with *Arabidopsis* genomic DNA fragments for binding followed by pull-down and purification of the fragments bound to the TFs. The purified fragments were sequenced by a next-generation sequencer to identify genome-wide physical binding sites of the TFs.

This analysis identified a total of 21,710 merged binding sites of homodimers of the seven bZIP TFs in the *Arabidopsis* genome (Table 1). They include non-promoter binding sites that were seen in coding sequences, 3′ untranslated regions and intergenic regions. We detected large overlaps of physical binding sites between HY5 and GBF2/GBF3. Of the HY5-binding sites, 53.0%, 40.3%, 82.4% and 86.7% overlapped with those of HYH, GBF1, GBF2 and GBF3, respectively (Figure S5). Furthermore, in the promoter regions of the 863 HY5-binding sites, 60.0%, 43.8%, 84.7% and 88.9% overlapped with those of HYH, GBF1, GBF2 and GBF3, respectively (Figure 5B). In particular, the binding sites of GBF2 and GBF3 largely covered those of HY5, indicating the possibility that these TFs interfere with HY5 function in either a positive or negative manner.

Binding motifs were predicted from gDB-seq and the prediction generated G-box sequences and the surrounding bases for all bZIP TFs tested (Figure S6). For example, the HY5-inducible *CRY3* gene promoter possesses a predicted binding site containing two G-boxes and this site was shared among bZIP TFs, including GBF2 and GBF3 (Figure 5C).

### 2.6. Physical Interaction of HYH, GBF1, GBF2 and GBF3 with HY5

**Figure 6:**
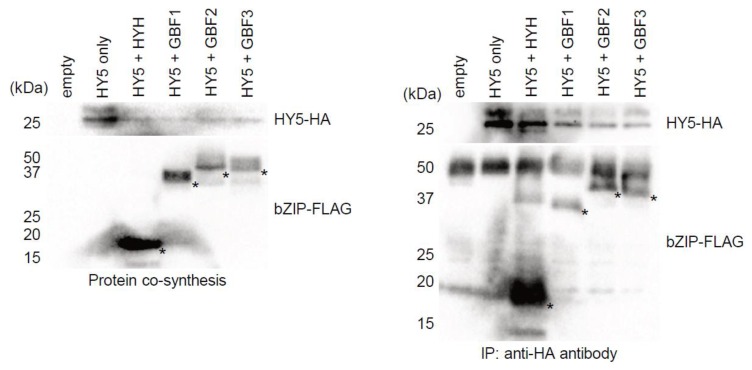
In vitro interaction between HY5 and other bZIP TFs. Detection of recombinant proteins by Western blot analysis before (**A**) and after (**B**) immunoprecipitation. HA-tagged HY5 was co-synthesized with FLAG-tagged HYH, GBF1, GBF2 or GBF3 proteins in wheat germ extract and then immunoprecipitated with anti-HA antibodies.

gDB-seq analysis provides the physical binding sites of only TF homodimers and not heterodimers. It is assumed that bZIP TFs function by forming heterodimers in vivo. To examine whether HY5 physically interacts with other bZIP TFs, HA-tagged HY5 proteins were co-synthesized with other FLAG-tagged bZIP TFs in a wheat germ extract and immunoprecipitated with anti-HA antibodies. The results showed that HYH, GBF1, GBF2 and GBF3 all co-immunoprecipitated with HY5 (Figure 6), indicating that they could form a heterodimer with HY5 and may modulate transcription of the target genes in vivo.

## 3. Discussion

Light is an essential factor for signaling towards proper morphogenesis as well as energy production in plants [1]. Higher plants mainly perceive blue, red and far-red light wavelengths through their respective photoreceptors [2]. Until now, there have been few reports that clearly describe a difference in gene expression changes following exposure to blue, red and far-red light from darkness. Here, we monitored them by RNA-seq analysis.

Perception of different light wavelengths can induce different physiological responses [2]. However, unique changes in gene expression upon exposure to each light wavelength, except to blue light, are limited (Figure 2), and the trend of the expression profiles under each light become similar by 12 h after exposure (Figure 1B). The reason for this is probably that signals from the different wavelengths converge into *HY5* at an early stage of the light response and *HY5* is responsible for the control of downstream gene expression. Indeed, early accumulation of *HY5* mRNAs reached the maximum level 1 h after exposure (Figure 4C). Apart from this, we observed a mild difference in the transcriptomic trend between blue and red/far-red at the early to middle stage of light exposure (Figure 1B,C). This difference is presumably derived from the fact that both red and far-red light pass through phytochromes and blue light is perceived by cryptochromes or phototropins [2].

GO analysis of independent DEGs in blue light showed that abiotic stress-related terms are enriched (Figure S2). This result may be due to the higher intensity of the blue light than the red and far-red light (see Materials and Methods); a previous report showed that excess blue light induced the generation of reactive oxygen species, characteristic of the abiotic stress response [26].

*HY5*, encoding a bZIP-type transcription factor, is a hub gene in the early light response [3]. Loss-of-function mutants show inhibition of photomorphogenesis, such as long hypocotyls and small cotyledons, when they are young seedlings [7,8,9,10,11]. Our previous transcriptomic analyses revealed that the response of some light-responsive genes to blue light exposure after darkness was reduced in *hy5* [6,23]. Although the number of DEGs that can be directly regulated by HY5 was limited in this analysis, many of them were shared among the three monochromatic wavelengths (Figure 3). This evidence supports the present notion that gene expression directly and indirectly controlled by HY5 could underpin photomorphogenesis [4,5].

In vitro co-synthesis of bZIP proteins and the immunoprecipitation experiment with HY5 showed that HY5 physically interacts with HYH, GBF1, GBF2 and GBF3 (Figure 6), indicating the possibility that they could form heterodimers with HY5. Previous studies have reported that, by forming heterodimers with HY5, GBF1 and HYH modulate their binding properties compared with those of HY5 homodimers [15] and negatively and positively, respectively, regulate hypocotyl elongation under light [9]. Our time-course transcriptome analysis shows that accumulation of *HYH* mRNAs was induced by light exposure in a similar manner to *HY5* but reached a maximum level at 3 h, later than that of *HY5* which reached a maximum at 1 h (Figure 4C). In addition, accumulation of *GBF3* mRNAs was also induced in a similar manner to *HY5*, and co-expression analysis with *HY5* showed that the expression transition of *GBF3* and *GBF2* was more similar to that of *HY5* than that of *HYH* (Figure 4B and Figure S4). From these results, it is speculated that, for example, GBF3 also affects HY5-binding properties by forming a heterodimer with HY5 in vivo and participates in the early light response. As GBF2′s amino acid sequence is similar to GBF3 (Figure 4A) and GBF2 moves from the cytoplasm to the nucleus upon blue light exposure after darkness [17], it is possible that GBF3 moves in the same way.

A recent report showed that a bHLH-type TF positively regulates anthocyanin biosynthesis through forming a complex with HY5 in strawberry [27]. Thus, other factors like different types of TFs from bZIP-type TFs might modulate the HY5-binding property.

Previous work reported that a GBF4 homodimer synthesized in vitro by a rabbit reticulocyte lysate system did not bind to DNA, irrespective of the existence of a G-box-binding domain [24]. However, this gDB-seq study shows that GBF4 homodimers produced in wheat germ extract bind to gDNA fragments (Table 1) and the G-box sequence was actually predicted as a DNA-binding motif of GBF4 (Figure S6). Besides, GBF2, GBF3 and EEL bind to a much greater number of genomic sites and a G-box is predicted as their binding motif (Table 1). These results indicate that each bZIP TF might prefer specific peripheral sequences around the G-box in order to bind to DNA.

It is easy to speculate that multiple TFs orchestrate to act in an environmental response, such as that of light, forming a TF network [20,21,28,29]. Some kinds of TFs, such as bZIPs and bHLHs, bind to DNA, forming dimers. Although gDB-seq and DAP-seq are high-throughput methodologies to identify physical binding sites of TFs in vitro, they can detect genomic sites that bind only to homodimers and not to heterodimers. On the other hand, ChIP-seq can detect genomic sites bound to both dimers, but it is hard to distinguish the two at present. This may partly explain the limitation of the overlap of the HY5 targets identified between ChIP-seq and gDB-seq (Figure 3A). Therefore, establishment of a novel methodology to identify heterodimer binding sites and the effect on phenotype will contribute to the expansion of our knowledge on dynamic TF networks.

In this report, we provide a transcriptomic resource following monochromatic light irradiation after darkness in young *Arabidopsis* seedlings, and information on the genome-wide physical binding sites of homodimers of seven bZIP TFs including light-regulating TFs as well as other candidates. The transitions of gene expression profiles downstream of light perception involve complicated regulation orchestrated by various TF networks. We hope that our results will contribute to revealing the whole picture of the regulatory mechanism of gene expression change upon light irradiation.

## 4. Materials and Methods

### 4.1. Plant and Light Exposure

Wild-type *Arabidopsis thaliana* ecotype Col-0 was used in this research. Procedures of plant growth and light exposure have been described previously [6,23,30]. Seedlings grown for 3 days in the dark at 22 °C were exposed to white (80 μmol m^−2^ s^−1^), blue (24 μmol m^−2^ s^−1^), red (9 μmol m^−2^ s^−1^) and far-red (1.25 μmol m^−2^ s^−1^) light. The seedlings were harvested at 0, 1, 3, 6 and 12 h after light exposure.

### 4.2. RNA-Seq Analysis

Total RNA was extracted from the harvested seedlings using the TRIzol reagent (Thermo Fisher Scientific, Waltham, MA, USA). RNA-seq libraries were constructed using a TruSeq Stranded mRNA Library Prep Kit (Illumina, Inc., San Diego, CA, USA) and pair-end sequenced using a HiSeq 2000. The sequenced reads of time-course RNA-seq analysis upon light exposure were mapped onto the *Arabidopsis* TAIR10 genome using TopHat [31] after excluding rRNAs/tRNAs. FPKM values of protein-coding genes were calculated using Cufflinks [32]. Two biological replicates were performed for each light exposure. Significant differences were defined by a 2-fold change (light vs. dark) and the Benjamini–Hochberg method (*q*-value < 0.05).

Gene ontology enrichment analysis was performed using a TAIR tool (https://www.arabidopsis.org/tools/go_term_enrichment.jsp). GO terms were obtained from the GO Ontology database released on 2019-10-08 by the Fisher’s Exact test using the Bonferroni correction for multiple testing (*p* < 0.05).

The R/Bioconductor package cummeRbund [32] was used to find bZIP genes that showed a similar expression profile to *HY5*.

### 4.3. gDB-seq Analysis

Coding sequences for HA-tagged *HYH*, *GBF1*, *GBF2* and *GBF3* were amplified by PCR from full-length cDNA clones using gene-specific primers listed in Table S3 and inserted into pEU-His vectors using appropriate restriction enzymes creating pEU-His-HYH-HA, pEU-His-GBF1-HA, pEU-His-GBF2-HA and pEU-His-GBF3-HA. A series of gDB-seq procedures including protein synthesis, immunoprecipitation and sequencing by MiSeq (Illumina, Inc., San Diego, CA, USA), mapping of sequenced reads onto the genome by Bowtie 2 [33] and peak detection by MACS2 (*q*-value < 0.01) [34] have been described previously [6]. For constructing the gDB-seq library, a KAPA Hyper Prep Kit (Roche, Basel, Switzerland) was used instead of a TruSeq ChIP Library Preparation Kit (Illumina, Inc., San Diego, CA, USA). Binding motif sequences were predicted from gDB-seq data using the GADEM program [35]. JBrowse was used for visualization of the peak position of gDB-seq [36]. Promoters were defined as regions from 500 nt upstream to 200 nt downstream of the 5′ ends of genes.

### 4.4. SDS-PAGE

For the detection of recombinant proteins used for gDB-seq, purified recombinant proteins were mixed with 2× SDS-PAGE sample buffer (0.125 M Tris-HCl pH 6.8, 4% SDS, 20% glycerol, 10% 2-mercaptoethanol and 0.01% bromophenol blue) and boiled at 95 °C for 5 min. The samples were run out on a 10–20% SuperSep Ace gel (FUJIFILM Wako, Osaka, Japan). The gels were stained with CBB One Super (Nacalai Tesque, Kyoto, Japan) for visualization.

### 4.5. Re-Analysis of HY5 ChIP-Seq

ChIP-seq with *HY5-YFP* transgenic *Arabidopsis* plants has been described previously [24]. The sequence data was re-processed in the same way as gDB-seq. Peak detection was performed using MACS2 (*p*-value < 0.05) [34].

### 4.6. Co-Synthesis and Immunoprecipitation In Vitro

Coding sequences for FLAG-tagged *HYH*, *GBF1*, *GBF2* and *GBF3* were amplified by PCR from full-length cDNA clones using gene-specific primers listed in Table S3 and inserted into the pEU-His vector using appropriate restriction enzymes, creating pEU-His-HYH-FLAG, pEU-His-GBF1-FLAG, pEU-His-GBF2-FLAG and pEU-His-GBF3-FLAG. Another expression vector, pEU-His-HY5-HA, has been described previously [6]. FLAG-tagged recombinant proteins (His-bZIP-FLAG) were co-synthesized with HY5-HA in a wheat germ extract using a WEPRO7240H Kit (CellFree Sciences, Yokohama, Japan), according to the manufacturer’s instructions. For the pre-wash, 120 ul of the crude extracts containing both His-HY5-HA and His-bZIP-FLAG was mixed with 330 μL of binding buffer (10 mM Tris-HCl pH7.5, 50 mM KCl, 5 mM MgCl_2_, 1 mM DTT, 0.05% Triton-X100, 2.5% glycerol) and 30 μL of Dynabeads protein G (Thermo Fisher Scientific, Waltham, MA, USA), followed by rotation at 4 °C for 2 h. Prior to immunoprecipitation, 5 μL of anti-HA antibody ((FUJIFILM Wako, Osaka, Japan) was conjugated to 30 μL of Dynabeads Protein G. The Dynabeads were mixed with a pre-washed mixture containing the recombinant proteins, rotated at 4 °C for 2 h, and washed three times with 1 mL of binding buffer. They were then resuspended in 50 μL of binding buffer, mixed with 15 μL of 4× SDS-PAGE sample buffer (Bio-Rad Laboratories Inc., Hercules, CA, USA) and boiled at 95 °C for 5 min. The samples were run out on a 10–20% SuperSep Ace gel (FUJIFILM Wako, Osaka, Japan) and blotted onto an Immobilon-P Transfer Membrane (Merck Millipore, Burlington, MA, USA). Anti-HA and anti-FLAG antibodies (FUJIFILM Wako, Osaka, Japan, 1:1000 dilution) and horseradish peroxidase-linked anti-mouse antibody (GE Healthcare, Chicago, IL, USA, 1:10,000 dilution) were used for the immune reactions. Proteins were visualized by Chemi-Lumi One Super (Nacalai Tesque, Osaka, Japan) and ChemiDoc XRS Plus (Bio-Rad Laboratories Inc., Hercules, CA, USA).

### 4.7. Data Deposition

The data set of sequenced reads by next-generation sequencers is deposited in the DDBJ/EMBL/GenBank BioProject under accession number PRJNA610701.

## Figures and Tables

**Table 1:** Numbers of binding sites of seven bZIP TFs.

	All Peaks	HY5	HYH	GBF1	GBF2	GBF3	GBF4	EEL
Binding site	21710	2083	1276	1340	11737	18410	2742	14716
Binding to promoter		863	565	531	2331	3043	1050	3024

## Supplementary Materials

Supplementary Materials can be found at https://www.mdpi.com/1422-0067/21/6/1993/s1.

## Abbreviations

bZIP	basic ZIpper Protein
gDB-seq	genomic DNA Binding sequencing
HY5	ELONGATED HYPOCOTYL 5
GBF	G-box Binding Factor
TF	Transcription Factor
DEG	Differentially Expressed Gene
