# Supplementary material for: Time-Course Transcriptome Study Reveals Mode of bZIP Transcription Factors on Light Exposure in Arabidopsis
Source: Int J Mol Sci. 2020 Mar 14;21(6):1993. doi: 10.3390/ijms21061993 (PMC7139404; doi:10.3390/ijms21061993)
Supplement: Supplementary file 1 [file ijms-21-01993-s001.zip › Supplementary_Figures.pdf]

Up-regulated

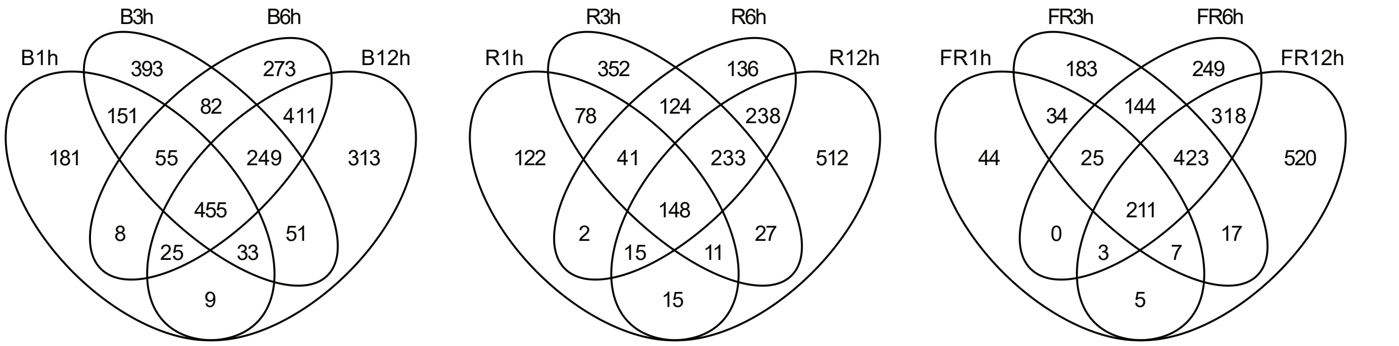

Down-regulated

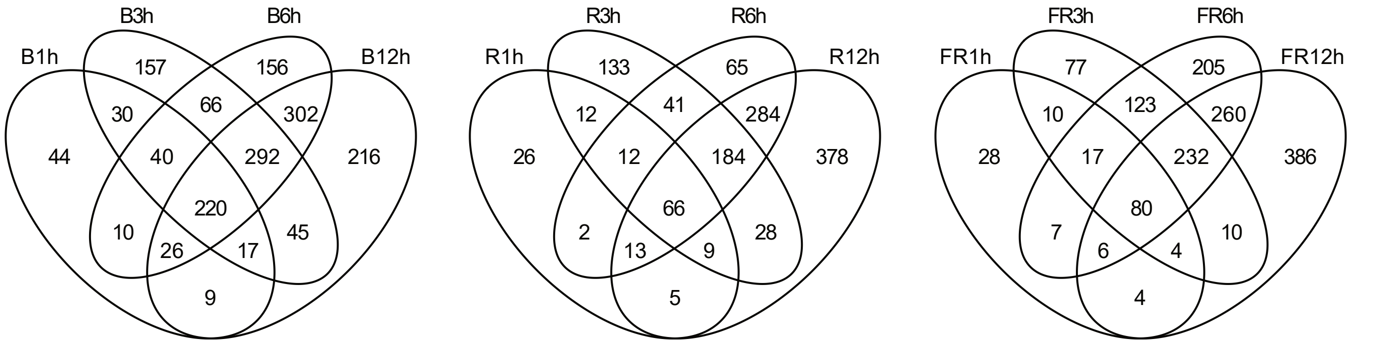

Figure S1 Overlap of up-regulated and down-regulated genes from four time points for each light exposure. B=Blue, R=Red, FR=Far-Red.

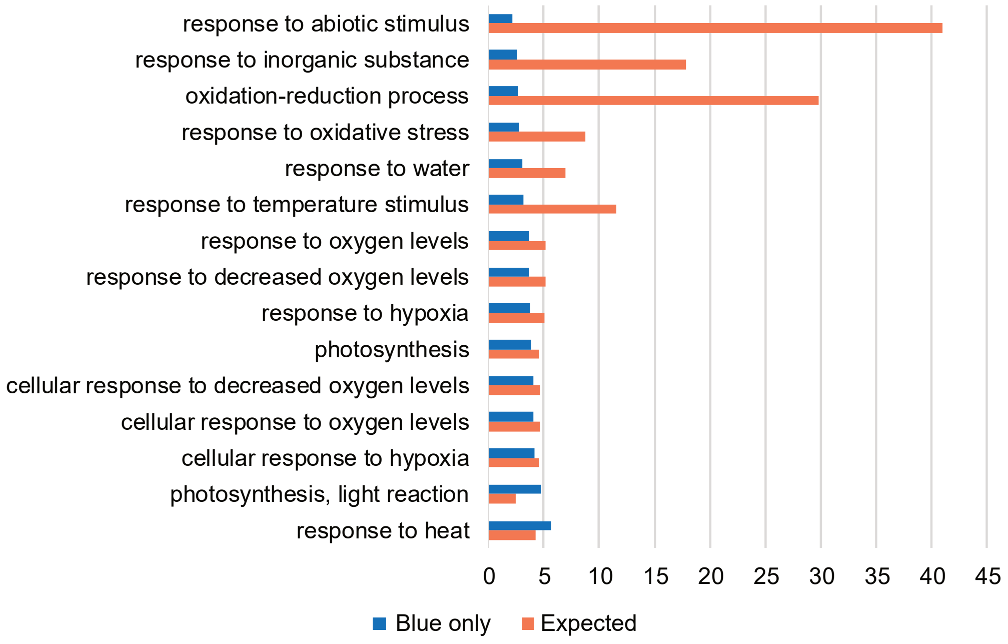

Figure S2 Gene ontology analysis of independent DEGs on blue light exposure.

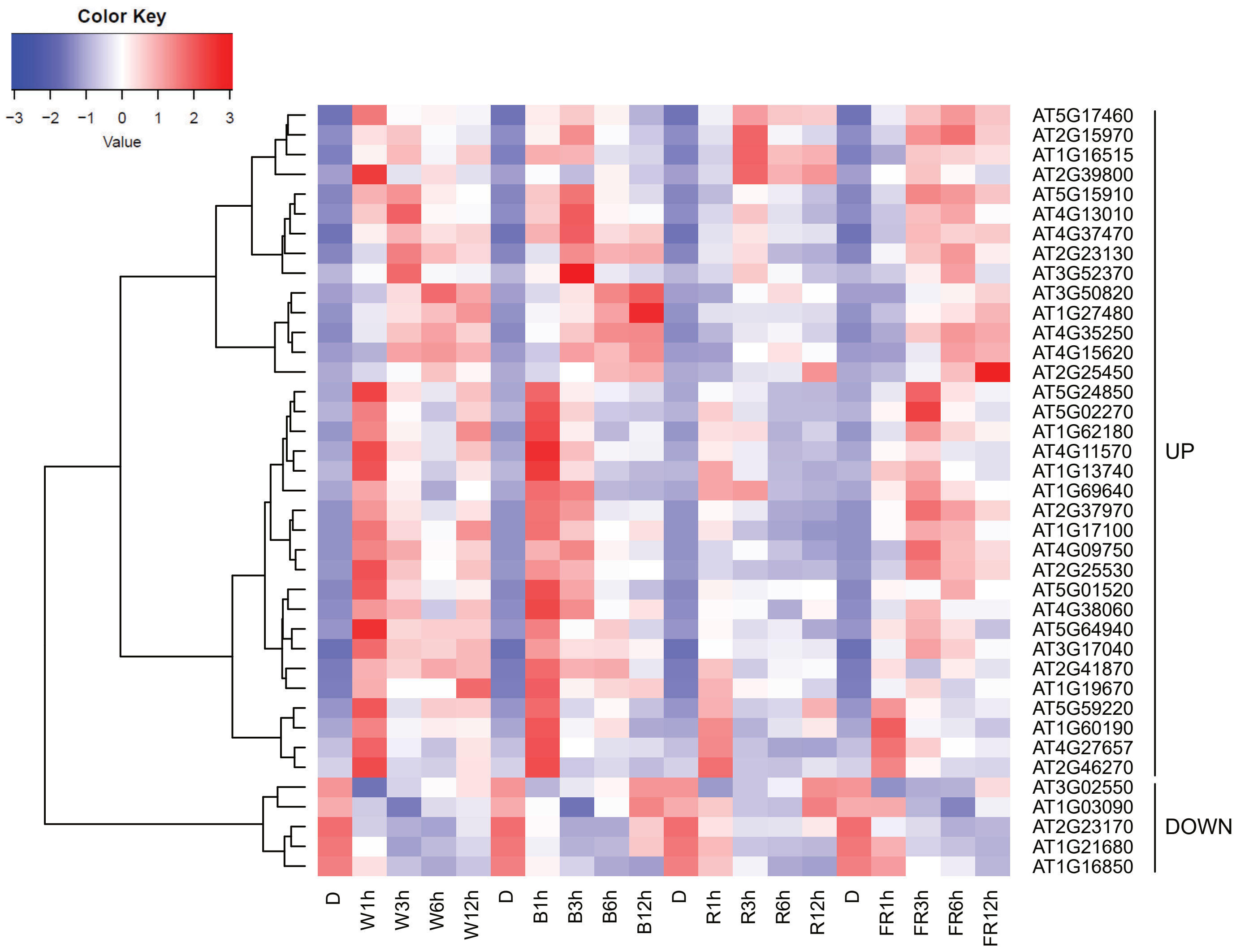

Figure S3 Heatmap (Z-score data) showing time-course expression of 34 up-regulated and five down-regulated genes, which overlapped with 328 HY5-binding genes identified in Figure 3A, on exposure to light. D=Dark, W=White, B=Blue, R=Red, FR=Far-Red.

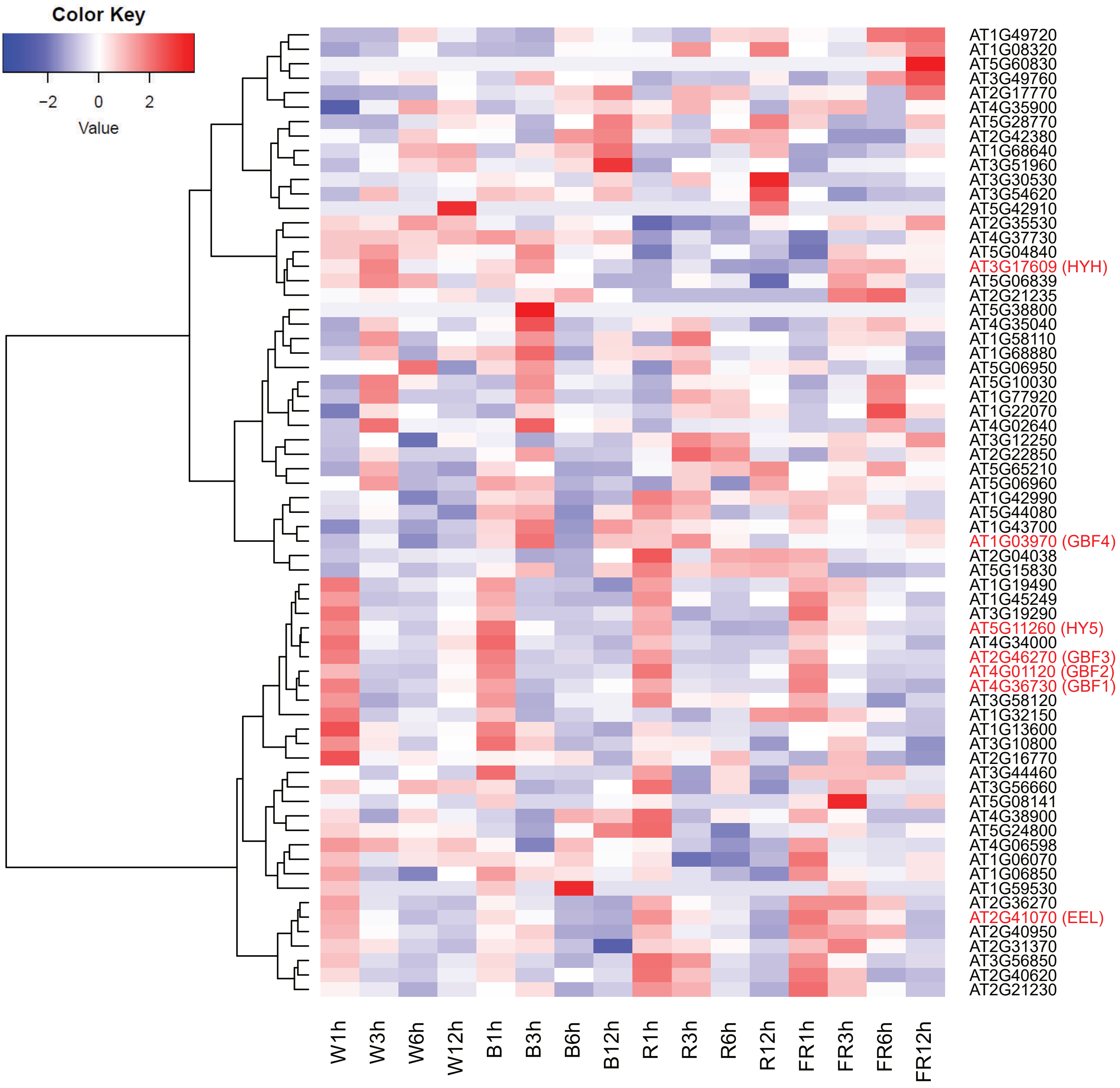

Figure S4 Heatmap (Z-score data) showing time-course fold changes (light/dark) of 67 genes detected encoding bZIP TFs on exposure to light. W=White, B=Blue, R=Red, FR=Far-Red.

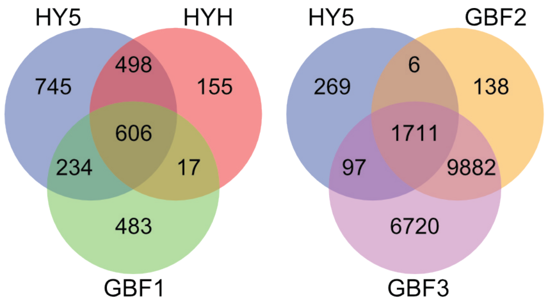

Figure S5 Overlap of physical binding sites identified in gDB-seq analysis between HY5, HYH and GBF1, and HY5, GBF2 and GBF3.

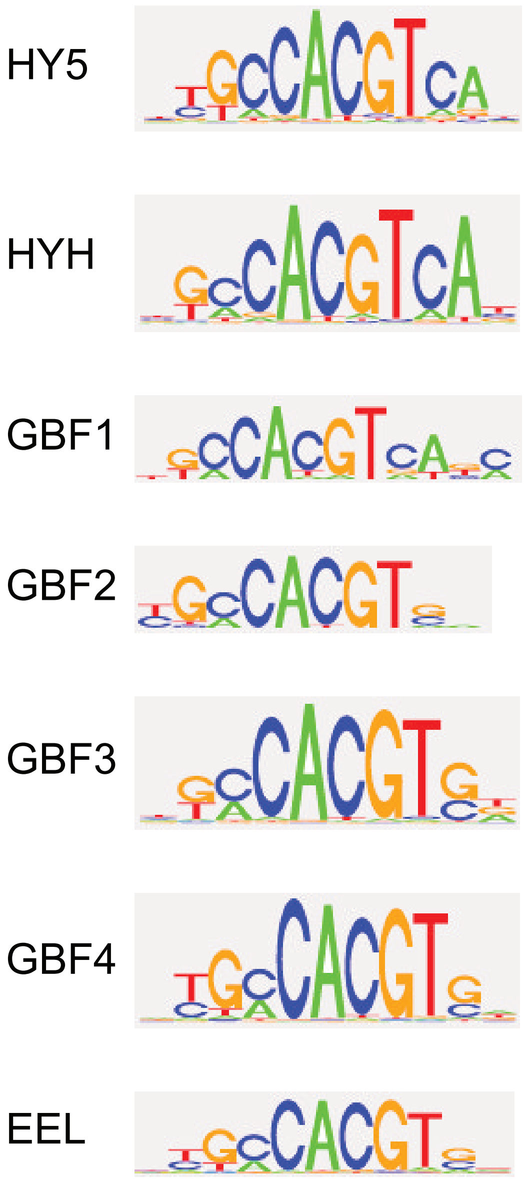

Figure S6 Predicted binding motif sequences of the seven bZIP TFs from gDB-seq analysis.
